# Supplementary material for: Psychosocial Features of Shift Work Disorder
Source: Brain Sci. 2021 Jul 14;11(7):928. doi: 10.3390/brainsci11070928 (PMC8306952; doi:10.3390/brainsci11070928)
Supplement: Supplementary file 1 [file brainsci-11-00928-s001.zip › brainsci-1263379-supplementary.pdf]

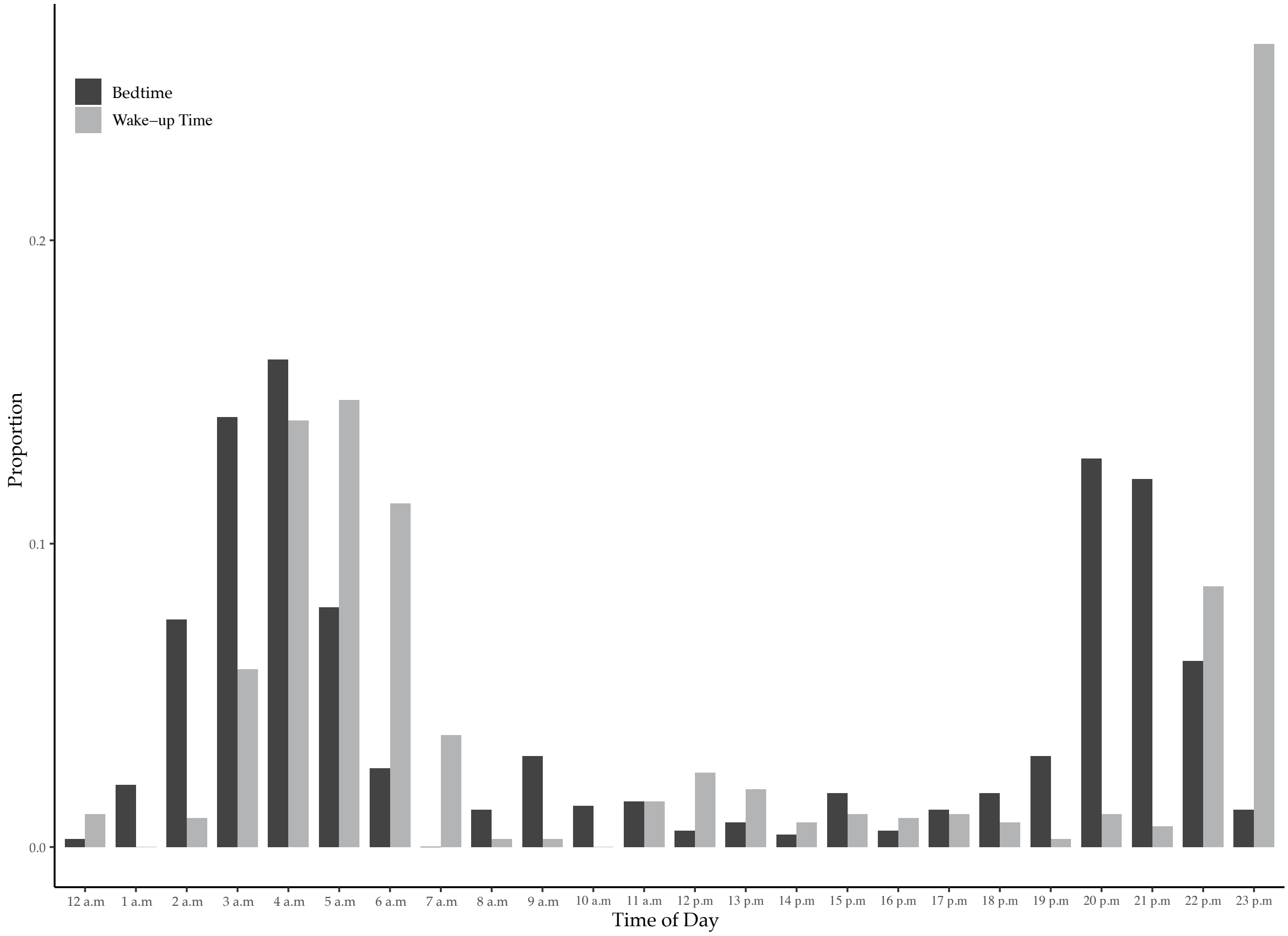

Supplemental Figure S1. The Distribution of Napping Over a 24−hour Period for Night Workers.

a)

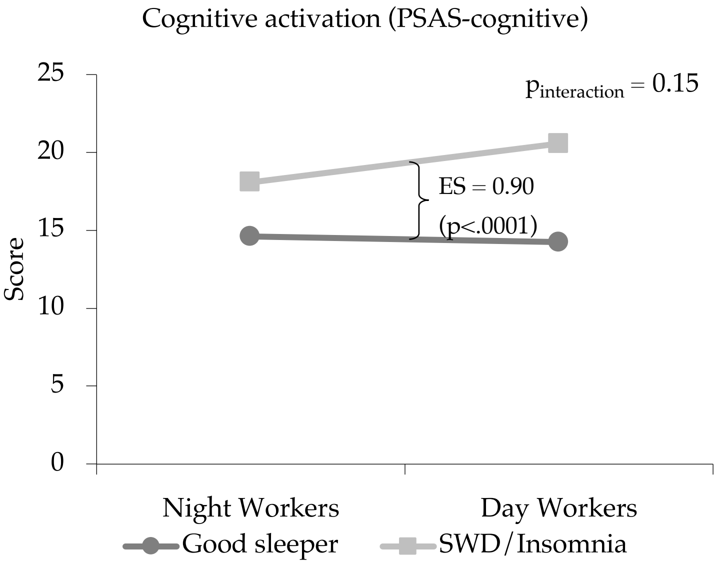

b)

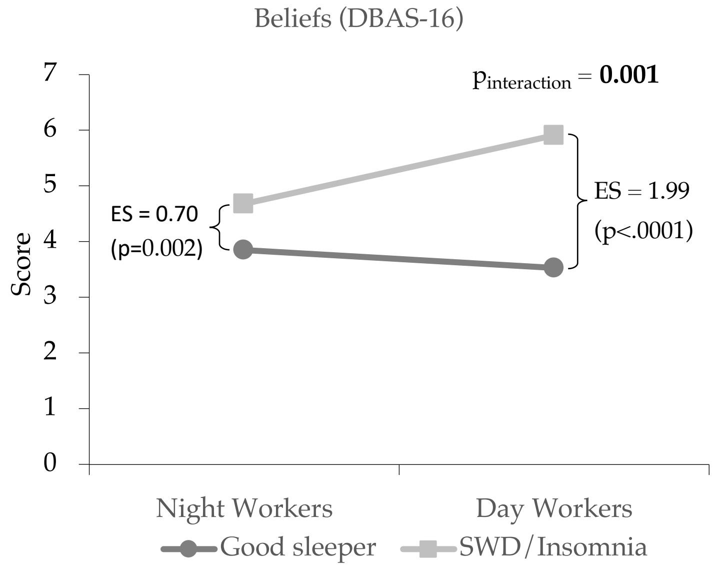

c)

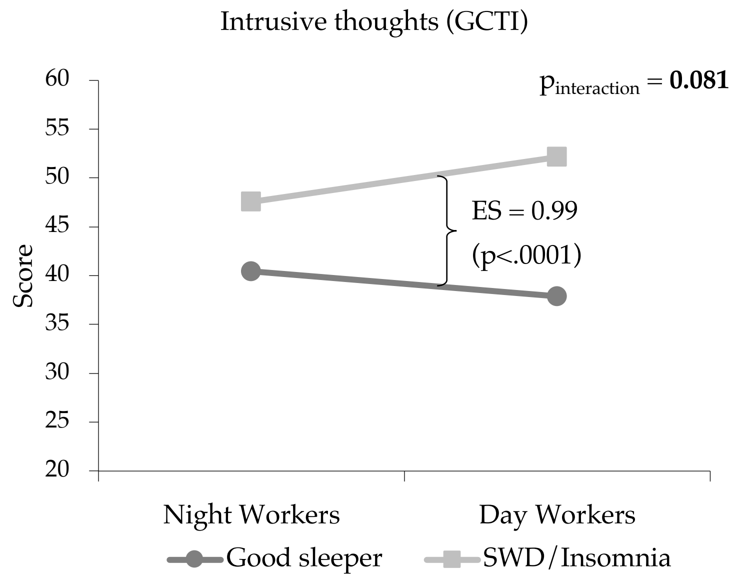

Supplemental Figure S2. Cognitive activation variables according to Work Schedule (Night or Day Workers) and Sleep Disorder (Good Sleep or SWD/insomnia).

Note. PSAS-cognitive = Predisposition Sleep Arousal Scale cognitive scale; DBAS-16 = Dysfunctional beliefs and attitudes about sleep 16-item; GCTI= Glasgow content of thoughts inventory; SWD = Shift Work Disorder; ES = Effect sizes.
